# Investigating Employees’ Concerns and Wishes Regarding Digital Stress Management Interventions With Value Sensitive Design: Mixed Methods Study

**DOI:** 10.2196/44131

**Published:** 2023-04-13

**Authors:** Jasmine I Kerr, Mara Naegelin, Michaela Benk, Florian v Wangenheim, Erika Meins, Eleonora Viganò, Andrea Ferrario

**Affiliations:** 1 Mobiliar Lab for Analytics at ETH Zurich Department of Management, Technology, and Economics ETH Zurich Zürich Switzerland; 2 Chair of Technology Marketing Department of Management, Technology, and Economics ETH Zurich Zurich Switzerland; 3 Institute of Biomedical Ethics and History of Medicine University of Zurich Zurich Switzerland

**Keywords:** value sensitive design, digital health intervention, stress, employee well-being, monitoring, machine learning, ethics, mobile phone

## Abstract

**Background:**

Work stress places a heavy economic and disease burden on society. Recent technological advances include digital health interventions for helping employees prevent and manage their stress at work effectively. Although such digital solutions come with an array of ethical risks, especially if they involve biomedical big data, the incorporation of employees’ values in their design and deployment has been widely overlooked.

**Objective:**

To bridge this gap, we used the value sensitive design (VSD) framework to identify relevant values concerning a digital stress management intervention (dSMI) at the workplace, assess how users comprehend these values, and derive specific requirements for an ethics-informed design of dSMIs. VSD is a theoretically grounded framework that front-loads ethics by accounting for values throughout the design process of a technology.

**Methods:**

We conducted a literature search to identify relevant values of dSMIs at the workplace. To understand how potential users comprehend these values and derive design requirements, we conducted a web-based study that contained closed and open questions with employees of a Swiss company, allowing both quantitative and qualitative analyses.

**Results:**

The values *health and well-being, privacy, autonomy, accountability,* and *identity* were identified through our literature search. Statistical analysis of 170 responses from the web-based study revealed that the intention to use and perceived usefulness of a dSMI were moderate to high. Employees’ moderate to high health and well-being concerns included worries that a dSMI would not be effective or would even amplify their stress levels. Privacy concerns were also rated on the higher end of the score range, whereas concerns regarding autonomy, accountability, and identity were rated lower. Moreover, a personalized dSMI with a monitoring system involving a machine learning-based analysis of data led to significantly higher privacy (*P*=.009) and accountability concerns (*P*=.04) than a dSMI without a monitoring system. In addition, *integrability*, *user-friendliness*, and *digital independence* emerged as novel values from the qualitative analysis of 85 text responses.

**Conclusions:**

Although most surveyed employees were willing to use a dSMI at the workplace, there were considerable *health and well-being* concerns with regard to effectiveness and problem perpetuation. For a minority of employees who value *digital independence*, a nondigital offer might be more suitable. In terms of the type of dSMI, *privacy* and *accountability* concerns must be particularly well addressed if a machine learning-based monitoring component is included. To help mitigate these concerns, we propose specific requirements to support the VSD of a dSMI at the workplace. The results of this work and our research protocol will inform future research on VSD-based interventions and further advance the integration of ethics in digital health.

## Introduction

### Background

The prevalence of work stress has been increasing over the last decades [1-3], and its detrimental effects on both physical and mental health are well established [4,5]. Recently, the global COVID-19 pandemic has resulted in an alarming rise in psychological stress and associated depressive symptoms [6,7], which calls for effective action. Digital health solutions, such as smartphone and web-based applications, have been increasingly introduced to address the prevention and management of stress [8-10], promising low costs, scalability, and high degrees of personalization [11,12]. However, the development of digital health and well-being technologies is inherently accompanied by an array of risks and concerns arising from the possible violation of human values—values that should guide their design and implementation [13-15].

### Digital Stress Management Interventions

Work stress is defined as a reaction to an imbalance between an individual’s resources and physical, psychological, social, and organizational demands [16]. Work stress is also frequently used interchangeably with occupational stress, job stress, and work-related stress [17]. In this work, we will focus on stress management interventions (SMIs) at the workplace that target individuals’ ability to cope with work-related stress (ie, secondary interventions [17]).

Over the last decades, a wide range of such SMIs (eg, cognitive behavioral and relaxation techniques) that prevent and counter the effects of stress have been developed [17-20]. More recently, advances in web, mobile, and sensor technologies have enabled the development of *digital* SMIs (dSMIs), which leverage digital technologies for some or all intervention components [8,21]. To enable personalization, digital health interventions often integrate large amounts of health-related data with the help of machine learning (ML; for a review, refer to the study by Triantafyllidis and Tsanas [22]). Researchers have proposed that delivering an intervention right when it is needed and adaptable to the user’s current context and state could be substantially beneficial [23,24]. Such interventions are referred to as just-in-time adaptive interventions (JITAIs) and generally rely on the help of ML to continuously predict the user’s current state (eg, stress levels) through the unobtrusive monitoring of physiological, behavioral, or contextual data sources [25-29] and to trigger intervention prompts at optimal opportune moments [23].

### The Ethics of Digital Health Technologies

With respect to the ethical risks accompanying new digital health (and well-being) technologies, research has shown that violations of human values may arise owing to (1) the use of new methods, such as artificial intelligence (AI) and ML; (2) the generation of big data through interaction with the technologies; (3) the emergence of new stakeholders (technology giants, start-ups, and citizen scientists) in the digital ecosystem; and (4) the lack of regulatory standards [15,30]. In this context, human values (in short: values) are defined as what “a person or group of people consider important in life,” [31] “guide [peoples’] choices by evoking a sense of basic principles of right and wrong,” provide a “sense of priorities, and create a willingness to make meaning and see patterns” [32]. Examples of the consequences of the violation of values in the context of digital health are the exposure or untransparent management of inherently sensitive biomedical big data (eg, not disclosing surveillance [33]), prevention of users from making informed and autonomous decisions about their own health, and shift of the responsibility of treatment from health professionals to patients and their caregivers [34]. Regarding the use of AI in digital health, researchers have been extensively discussing several values, such as *privacy, integrity, accountability,* and *transparency* [35,36], and the ethical challenges emerging from their violation. For example, the interpretability and fairness domains of ML research attempt to understand and appropriately manage the specific ethical concerns raised by the use of ML models [37,38]. These concerns include, for example, the use of black box models for high-stakes decision-making (eg, in legal justice system and health care) [39] and the promotion or perpetuation of discrimination through biased predictions [40,41].

To mitigate ethical risks and improve uptake and impact, researchers have started to advocate for a more patient- or user-centric approach to digital technologies [42-46]. This understanding acknowledges the inherent sensitivity of health-related big data as well as the empowerment of patients or users [47,48]. However, the ethical dimension is still frequently neglected in the design process and evaluation of digital health interventions [12,34,49].

### Value Sensitive Design

Value sensitive design (VSD) is a prominent approach developed to overcome this gap in system design and research [50]. VSD is a theoretically grounded framework that front-loads ethics by accounting for values throughout the design process of a technology [50,51]. More practically, VSD provides a methodology for the conceptual, empirical, and technical investigations of system design that are performed in an iterative and integrative manner [50,52]. Specifically, during the (1) conceptual investigation, VSD aims to identify and define relevant values in the context in which the technology will be used. Then, during the (2) empirical investigation, stakeholders in that context are directly involved in assessing their understanding of the identified values. Finally, the (3) technical investigation is responsible for the actual translation of values and norms into specific design requirements of the technology [53]. The VSD methodology helps identify value-related concerns and wishes emerging from users’ perception of a technology and thus fosters the acceptance and use of the technology by promoting its alignment with users’ values and expectations [50,52]. VSD has recently been applied in digital health contexts, such as in the development of a digital assistant for physiotherapeutic treatments used at home [54] and the design of a preventive health check app [55]. In the human-computer interaction domain, researchers have suggested VSD as a framework to guide the understanding of AI as a sociotechnical system and the measurement of trust in human-AI interactions [56].

However, despite the urgency of addressing the ethical concerns that arise from the use of digital health technology [13-15,57,58] and the need to manage stress at the workplace [59], to the best of our knowledge, no attempt to use VSD for dSMIs has yet been made. Therefore, it is still unclear which values are relevant for the design of dSMIs at the workplace or how users comprehend them—that is, whether and how much the values are important to them. It is also unclear whether the presence of a JITAI component in the intervention may affect users’ comprehension of the values, for example, owing to the distinct ethical concerns raised using ML models. As a result, actionable recommendations for the VSD-informed design of dSMIs at the workplace are still lacking.

### Goal of This Work

In this study, we used the VSD framework to answer the following research questions (RQs):

- RQ1: What relevant values can be identified from the literature in the context of a dSMI at the workplace?
- RQ2: How do potential users comprehend the identified values of a dSMI at the workplace with and without a JITAI component?
- RQ3: What other values are raised by users that were not identified through the literature search?

To do so, we followed the 3-step approach of VSD. First, we identified potential users’ values based on the literature. Second, we assessed potential users’ perspective on value-related concerns and wishes regarding a dSMI at the workplace using a mixed methods web-based study. In particular, we investigated whether value comprehension differs across user groups based on sample characteristics and an experimental manipulation (JITAI dSMI vs non-JITAI dSMI) and whether any additional, novel value-related concerns and wishes are raised by the participants in the study. Finally, we drew from the value comprehension to inform specific VSD requirements for the development and deployment of a dSMI at the workplace.

## Methods

### Literature Search: Identifying Values

We conducted a narrative literature search to identify relevant values in the context of designing and deploying a dSMI at the workplace (conceptual investigation in VSD; refer to Multimedia Appendix 1 for detailed information on the search [15,31,34,35,44,49,54,58,60-75]). These values were identified using a 2-step process. In the first step, 2 researchers independently scanned the set of retrieved articles for desires, attitudes, beliefs, concerns, and needs discussed in the realm of using technology and advanced analytics for the purpose of health promotion and disease management both in and out of the workplace. In the second step, these constructs were synthesized and clustered into common human values that are specifically relevant for the design, development, and deployment of a dSMI at the workplace.

### Web-Based Study: Comprehending Values

#### Participants and Recruitment

As we planned to investigate the differences along sample characteristics and conditions, an a priori power analysis with G*Power for a 2-sided independent samples *t* test with a medium effect size (Cohen *d*=0.5), an α of .05, and power of 0.95 was performed, which revealed a sample size of 210 (105 per condition). Participants were recruited via a news post on the intranet of a large Swiss insurance company. The company offers insurance products to private individuals and other companies and has national and international partnerships. As such, the company offers a wide range of positions, including insurance brokers, actuaries, risk and asset managers, administrative and IT staff, user experience designers, and human resource personnel. We aimed to target employees who mostly work digitally, for whom a dSMI would be the most easily implemented. The inclusion criteria were being employed by the respective company, being aged between 18 and 65 years, and working on a computer for most working hours. These inclusion criteria could potentially be met by employees working as managers, professionals, technicians, associate professionals, or clerical support workers (ie, major groups 1 to 4 of the International Standard Classification of Occupations–8). The recruitment post was on the web for 12 days in August 2021. In total, 241 participants completed the questionnaire.

#### Procedure

**Figure 1:**
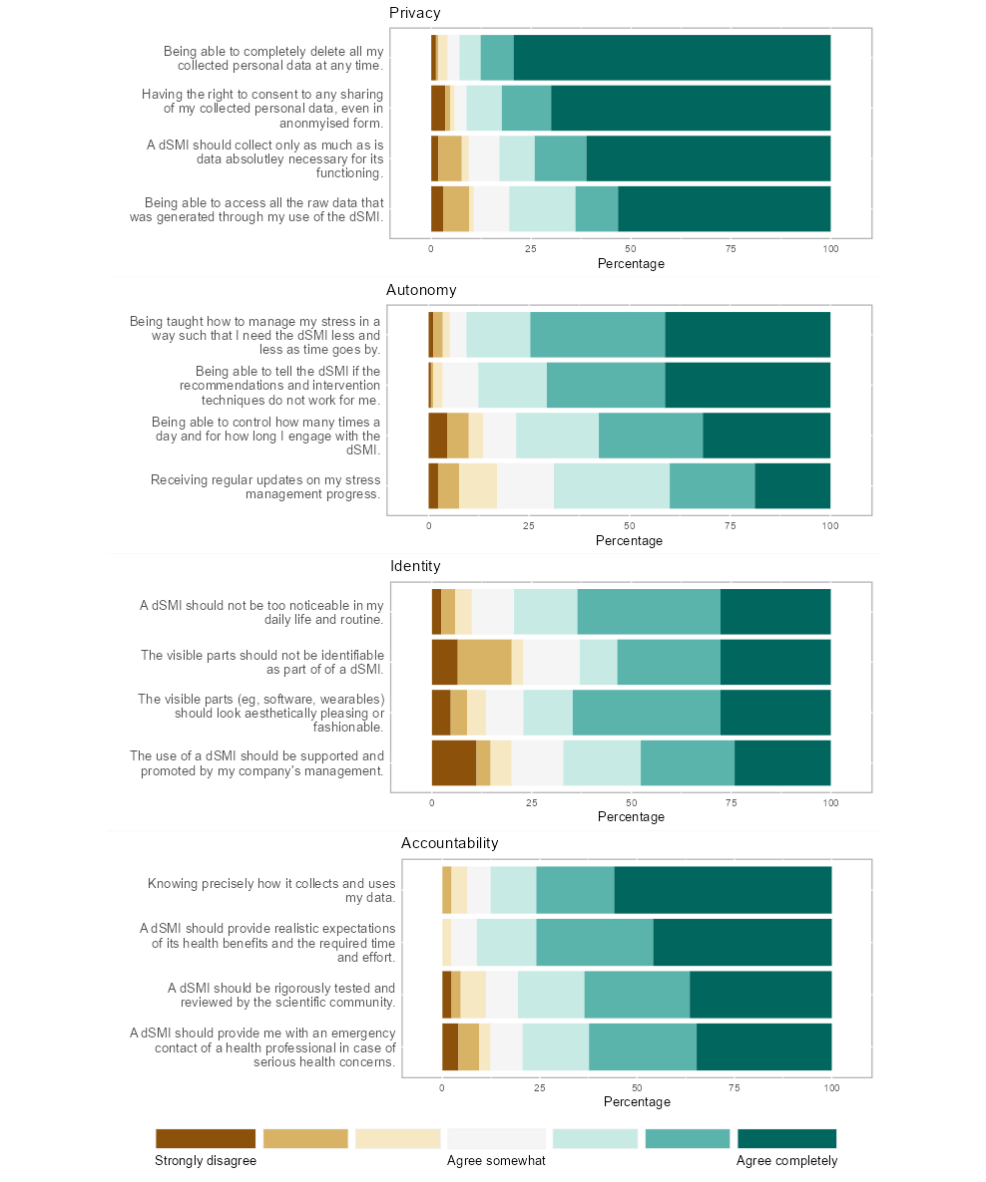
Value-related wishes. Responses in percentages to individual items reflecting a specific value. dSMI: digital stress management intervention.

**Figure 2:**
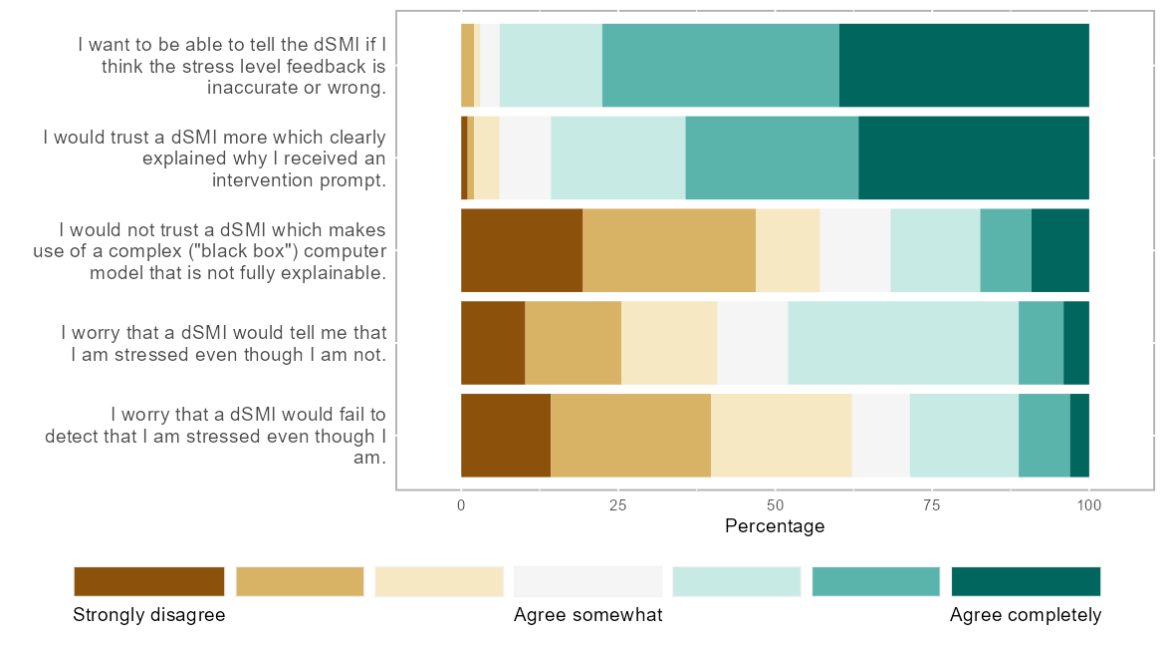
Just-in-time adaptive specific wishes. Responses in percentages to individual items regarding a digital stress management intervention (dSMI) with a just-in-time adaptive component.

The recruited participants were forwarded to a website hosted by the university, where they took part in the anonymous web-based study for approximately 15 minutes. Specifically, the participants were informed before participation that no identifiable information would be collected and that their IP address would not be recorded. First, the participants were asked to answer a set of initial questions (ie, sample characteristics). Then, the participants were asked to watch a 3-minute long video that introduced the general purpose, features, requirements, and benefits of a dSMI to establish a common understanding of the concept. There were 2 versions of the video, and the survey provider was programmed to randomly assign participants to one of them. One condition introduced a dSMI that included a monitoring system that was based on the user’s physiological and behavioral data and thus enabled JITAI prompts (JITAI dSMI condition), whereas the other condition introduced a dSMI without any monitoring system or JITAI components (non-JITAI dSMI condition). In all other aspects, the 2 presented dSMI versions were identical. Immediately after the video, the participants were asked to rate user acceptance–related items regarding the dSMI they were introduced to (refer to Table S1 in Multimedia Appendix 2 [76-78]). These questions were followed by self-developed scales tailored to reflect value-related concerns (Table S2 in Multimedia Appendix 2). Following these scales, the participants rated items reflecting value-related wishes (refer to Figures 1 and 2). Finally, the participants could share additional wishes and concerns in the form of free text, which would be analyzed qualitatively.

#### Independent Variables

##### Conditions

Both videos first taught the participants about the sources of stress and effects of stress on their mental and physical health. Then, they introduced the participants to the concept of dSMIs at the workplace, which are technology-supported interventions used at the workplace to manage stress. However, it was not specified who in the company would provide such an interventions. We also did not mention how data would be stored or handled or whether anybody from the company or an external party would have access to the data. These were details that we wanted to question the employees about after they watched the videos. Generally, a dSMI was explained as an application with various functions used on a computer, tablet, or smartphone. In the non-JITAI dSMI condition, the participants were then told that such an application could contain psychoeducation about stress, its causes, and its effects. Most importantly, the participants were told that a dSMI could offer a range of SMIs, such as guided relaxation techniques; teach cognitive behavioral strategies; be a coach for physical activity; offer biofeedback; provide nutrition advice; and teach time management techniques. Moreover, they were told that they could self-report their current stress level to track their stress levels and stress management progress. The participants in the JITAI dSMI condition received the same information with the addition of the stress detection component. The video explained how a dSMI could offer personal feedback on their current stress level based on bodily data such as cardiac activity or physical activity, which can be measured using not only wearables or their computers, including keyboard stroke dynamics or mouse movements, but also their subjective assessment of their stress level. The computation of their stress level would be done with ML algorithms. Finally, they were informed that this personalized feedback could be used for intervention prompts at opportune moments and to help track their stress management progress.

To assess whether the 2 versions of the video successfully conveyed the concept of a dSMI as well as the distinguishing aspects of the 2 conditions to the participants, we included manipulation check questions in the survey. Specifically, the participants were asked whether a dSMI as presented in the video (1) relied on digital technologies to help them manage their stress levels (true for both conditions) and (2) would collect data about their cardiac activity or computer mouse movements (true only for the JITAI dSMI condition).

##### Sample Characteristics

In addition to sociodemographic questions, the participants’ *levels of stress* were measured using the stress subscale of the Depression, Anxiety, and Stress Scales (DASS) [79,80], which consists of 7 items on a 4-point Likert scale. The aggregated score can be categorized into “normal,” “mild,” “moderate,” “severe,” and “extremely severe” levels of stress. The participants were also asked whether they already had *experiences with health technologies* (EXP) for the purpose of supporting relaxation, stress management, mental health, physical health, or fitness and physical activity. Finally, the participants rated their *propensity to trust health technologies* (PtT) using 3 items on a 5-point Likert scale. This scale was adapted from the Propensity to Trust Scale by Cheung and To [81]. PtT has been split into “low” and “high” PtT [82,83] based on a median split. Along with the experimental condition, these sample characteristics served as independent variables to test for differences in the dependent variables.

#### Dependent Variables

**Figure 3:**
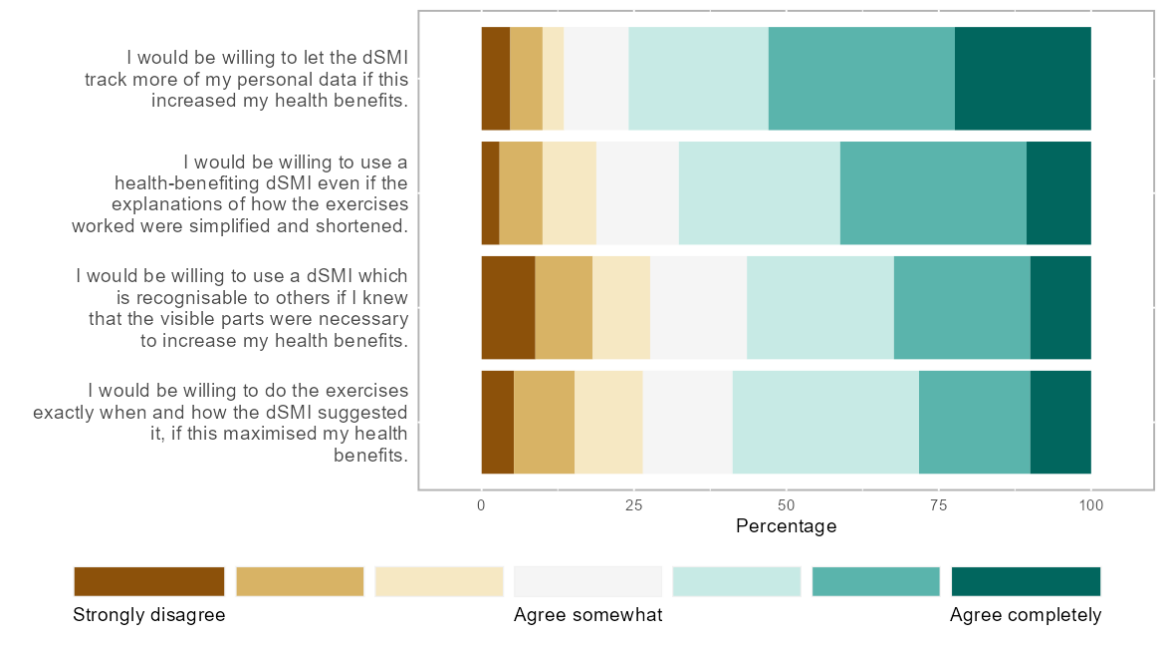
Percentages of willingness to trade the values (1) privacy, (2) accountability, (3) identity, and (4) autonomy for the value health and well-being. dSMI: digital stress management intervention.

**Figure 4:**
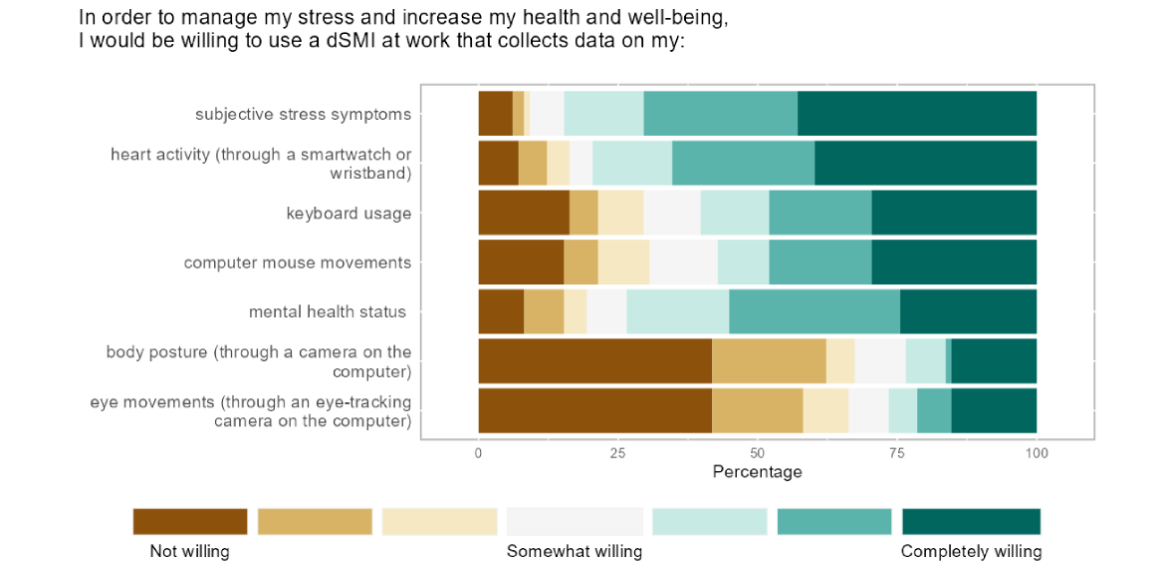
Percentages of willingness to share specific data sources for a digital stress management intervention (dSMI) with a just-in-time adaptive component.

As values are multifaceted and abstract constructs, we aimed to concretize them through specific value-related concerns or wishes to capture the value comprehension of the users. Per the identified value, we assessed *value-related concerns* and *value-related wishes* using 2 to 4 items each. Items reflecting value-related concerns were aggregated to 1 scale per value by taking the mean of all items, whereas items reflecting wishes were evaluated individually. All reflective indicators were derived from the literature and other existing items of related work (for the full battery of items, refer to Table S2 in Multimedia Appendix 2 and Figure 1). Moreover, we added 4 items aiming to capture value trade-offs (Figure 3). The participants in the JITAI condition were also asked specific questions regarding a dSMI that included a monitoring system (Figures 2 and 4). Finally, the closed questions included scales assessing constructs related to the user acceptance of technology (refer to Table S1 in Multimedia Appendix 2). All items of all closed questions were answered on a 7-point Likert scale. We performed a pretest of our survey with 38 participants, including researchers and laypeople, which helped reduce the number of items and length of the survey.

#### Statistical Analysis

Data analysis was performed using R (version 4.0.3; R Foundation for Statistical Computing) and RStudio (version 1.4.1103; RStudio Inc). A significance level of .05 was used for testing. Shapiro-Wilk normality tests of our models were significant in most cases, indicating violations of the assumption of normality of errors. The assumption of homogeneity of variances was also violated in some cases based on Levene tests. Thus, we ran nonparametric *t* and *F* tests to explore any differences along sample characteristics and the experimental manipulation. Significant omnibus tests were followed up with pairwise comparisons, which were adjusted using the Benjamini-Hochberg method. Cliff delta was used to compute effect sizes (negligible: |δ|<0.147; small: 0.147≤|δ|<0.330; medium: 0.330≤|δ|<0.474; large: 0.474≥|δ|).

#### Framework Method Analysis

We used framework method analysis (FMA) [84] to exploratively analyze the free-text data of the participants. FMA can be situated among the methods known as thematic analysis or qualitative content analysis and helps find similarities, differences, and relationships in the data clustered around themes. Asking the participants for free-text responses provided a way to extract further information on value-related wishes and concerns from them that might have been missed with the closed questions. In our case, 3 researchers first familiarized themselves with the data (ie, text from the open questions) and coded statements in the participants’ texts following FMA. As initial themes, they used not only the identified values but also the user acceptance–related variables. In addition, they scanned statements for new themes that did not fit the existing ones. Second, they developed a working analytical framework by identifying the most frequent themes in an iterative process. Third, they individually coded the data again with the agreed open framework. Fourth, they charted the coded statements into the framework matrix. Finally, they independently interpreted the data by identifying the key elements of all themes. These were then discussed jointly and in the presence of a fourth member of the research team naive to the data.

### Ethics Approval

This study was approved by the Swiss Federal Institute of Technology’s Ethics Committee (EK-2021-N-117).

## Results

### RQ1: Literature Search

#### Overview

The literature search (refer to Multimedia Appendix 1 for details of the search procedure), which was conducted as part of RQ1, generated 183 publications that were deemed relevant while sighting the titles and abstracts. Of these 183 publications, 24 (13.1%) were identified as relevant for determining relevant values after viewing their full texts (for the list of selected articles, refer to Multimedia Appendix 1). In our 2-step process, we identified five values that are specifically relevant for the design, development, and deployment of a dSMI at the workplace: (1) *health and well-being*, (2) *privacy*, (3) *autonomy*, (4) *identity*, *and* (5) *accountability*. In the next sections, we describe how and why we believe these values are relevant for digital health interventions and monitoring at the workplace.

#### Identified Values

A central value that emerged from the literature was *health and well-being*, which is sometimes summarized as human welfare and is viewed as a “fundamental intrinsic human value” [54]. According to the World Health Organization [85], health is seen “as a state of complete physical, mental and social well-being and not merely the absence of disease or infirmity” and is affected by biological, psychological, and social factors [86], whereas well-being is a state of positive mood and emotions being present, negative mood and emotions being absent, being satisfied with life, feeling fulfilled, and functioning in life [87-89]. Clearly, health and well-being are the main objectives of any digital health intervention [54]; therefore, they are an inherently important value for the design of a dSMI. If no other values are considered, any technological design could be conceivable if it improves health and well-being [54]. However, neglecting other values may not lead to or even compromise overall health and well-being in a person’s life [90]. Therefore, an intervention should follow the principles of nonmaleficence and beneficence [60]. Beneficence states that a treatment should benefit an individual’s health (eg, prevent and reduce stress levels), whereas nonmaleficence states that a treatment should do no harm (eg, not increase stress levels further).

Then, we identified *privacy*, which has been established as a central and fundamental ethical theme and frequently discussed in the context of digital health technologies and big data [49]. Privacy can be seen as a claim, an entitlement, or a right of an individual to determine what information about themselves can be communicated to others [91]. Personal privacy concerns the right to “private space” and the freedom of not being monitored or accessed [44]. Health-related data are inherently sensitive and can lead to stigmatization if exposed in any way [44]. As digital interventions in the field often work with technologies such as wearables, the obtrusiveness or visibility of such a device can lead to an infringement of personal privacy [44].

*Autonomy,* our third identified value, is viewed as a basic psychological need and deemed essential to overall health and well-being in the psychological self-determination theory [90]. Designing health interventions in an autonomy-supportive manner has been shown to increase long-term health and well-being [61,90]. As for autonomy, it can be defined as the freedom to choose and self-determine, the right to make personal decisions, and the right to independence [34,49,92-94]. For example, autonomy is not safeguarded if individuals feel pressured to use a dSMI or if they cannot make an informed decision because they are not transparently informed about the nature of the dSMI and any risks involved.

We then identified the value *identity,* which can be defined as people’s understanding of who they are over time, embracing both the continuity and discontinuity of the self over time [91]. A dSMI may become a placeholder for illness or an embodiment of illness, especially at the workplace, possibly increasing stigmatization and shaping both private and professional identity [34,44,49,95]. The identity of an individual also has a social aspect (ie, social identity), which includes holding a common social identification or being a member of a social group [96].

Finally, we identified the value *accountability*, which is defined as the ability to scrutinize judgments, decisions, and actions and holding decision-makers responsible for their consequences [35]. It is of great importance in the context of digital health, as health technology providers are not operating under professional codes of ethics that usually guide professional mental health therapists and clinicians and do not have to answer to ethics committees like researchers have to [62,97].

#### User Acceptance

Our literature search revealed that aside from the identified values or broader ethical discussions, researchers most frequently assessed users’ acceptance of digital health technologies [98-100]. Clearly, acceptance is a fundamental requirement for the adoption of and adherence to a dSMI. Therefore, we also included a set of user acceptance variables in our survey: *intention to use, perceived usefulness, trust,* and *distrust*. Here, we rely on the work by Ortega Egea and Román González [101], who extended the widely applied technology acceptance model [76,102] to add trust (as a predicting factor of intention to use) in the context of health information technology.

### RQ2: Web-Based Survey

#### Survey Development and Validation

Following the literature search and value identification, we developed closed questions based on the 5 identified values to address RQ2. Regarding the value *health and well-being*, which is the central objective of a dSMI, the concerns were split into 2 main aspects: concerns regarding beneficence (ie, not reducing stress) and concerns regarding nonmaleficence (ie, leading to increased stress [60]). As we aggregated the developed concern-related items into scales, we included a table containing a confirmatory factor analysis of these scales (Table S2 in Multimedia Appendix 2). In addition, we created 4 items to assess the degree to which the participants would trade the other 4 values for a greater health and well-being benefit from a dSMI. To assess the user acceptance of a dSMI, we included *trust*, *distrust*, *perceived intention to use*, and *usefulness* as additional dependent variables from the existing scales (refer to Table S1 in Multimedia Appendix 2).

#### Sample Characteristics

Overall, 241 participants took part in the web-based study; 3 (1.2%) responses had to be excluded for not passing the attention check, and another 62 (25.7%) responses had to be excluded for not passing the manipulation check. Finally, 2.5% (6/241) responses were excluded because of missing values. Therefore, 170 (70.5%) out of 241 responses (female participants: 71/170, 41.8%) were included in the final quantitative analysis of the data. For an overview of the sample characteristics, refer to Table 1.

**Table 1:** Characteristics of participants (n=170).

Characteristics			Participants, n (%)
**Age (years)**			
	18-29	28 (16.5)	
	30-39	51 (30)	
	40-49	38 (22.4)	
	≥50	53 (31.2)	
**Sex**			
	Female	71 (41.8)	
	Male	99 (58.2)	
**Position in the company**			
	Management	78 (45.9)	
	Employee	92 (54.1)	
**Propensity to trust health technologies**			
	Low	92 (54.1)	
	High	78 (45.9)	
**Experience with health technologies^a^**			
	Relaxation or stress management	40 (23.5)	
	Mental health	11 (6.5)	
	Physical health	9 (5.3)	
	Fitness and physical activity	133 (78.2)	
	None of the above	26 (15.3)	
**Level of stress (Depression Anxiety and Stress Scales)**			
	Normal	107 (62.9)	
	Mild	19 (11.2)	
	Moderate	29 (17.1)	
	Severe	13 (7.6)	
	Extremely severe	2 (1.2)	
**Condition^b^**			
	non-JITAI^c^ dSMI^d^	70 (41.2)	
	JITAI dSMI	100 (58.8)	

^a^Multiple responses possible.

^b^Represents the percentage of participants randomly assigned to 1 of the 2 conditions: a digital stress management intervention with a just-in-time adaptive component vs a digital stress management intervention without a just-in-time adaptive component.

^c^JITAI: just-in-time adaptive intervention.

^d^dSMI: digital stress management intervention.

#### Value-Related Concerns

**Figure 5:**
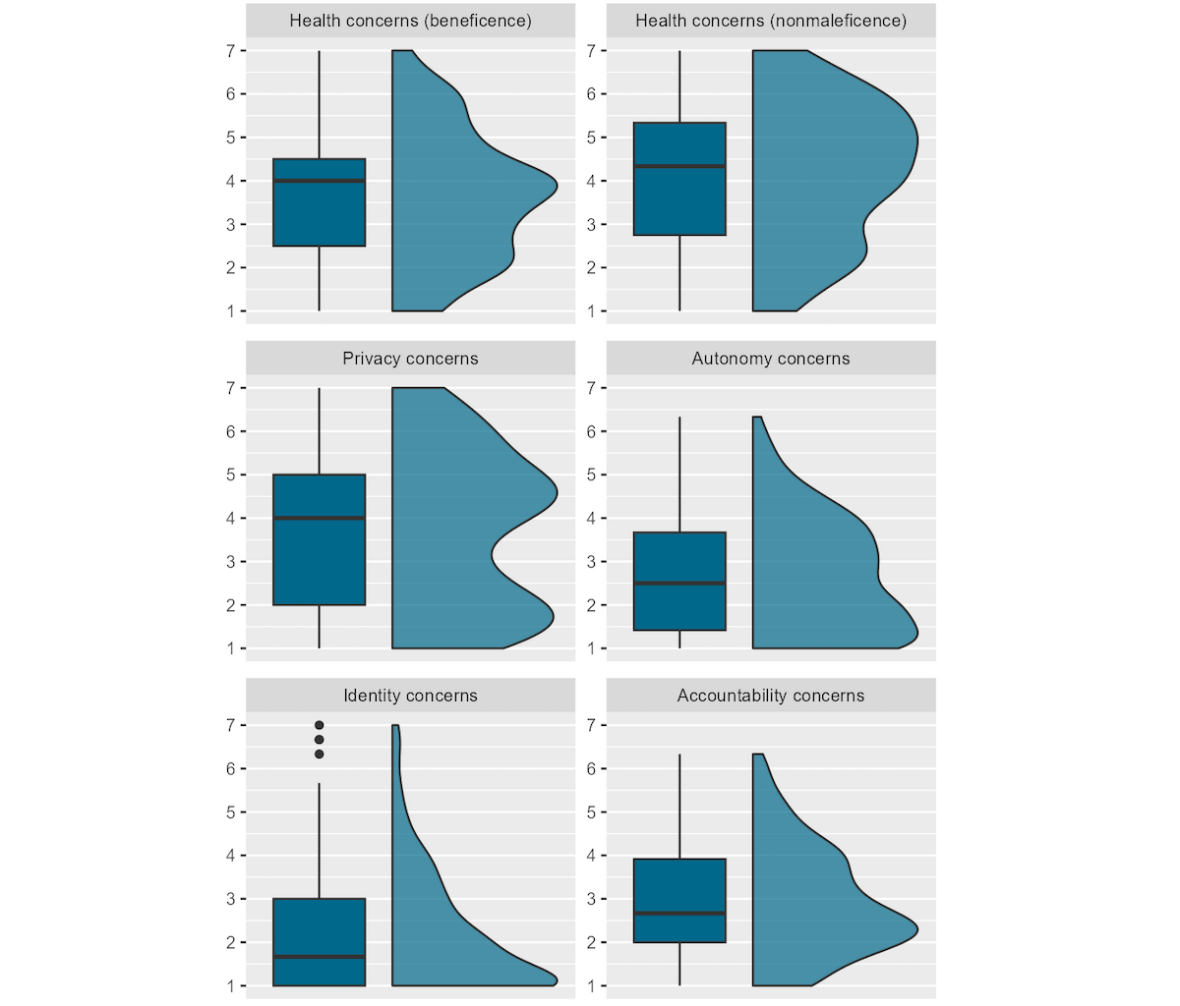
Box plots and distribution plots of value-related concerns.

The results showed that the mean levels of *autonomy* concerns (AUT; mean 2.63, SD 1.32), *identity* concerns (IDE; mean 2.14, SD 1.37), and *accountability* concerns (ACC; mean 2.91, SD 1.24) were situated in the lower range within the possible score range of 1 to 7, whereas participants’ *privacy* concerns (PRI; mean 3.69, SD 1.81) were moderate but with greater variability (see also Figure 5). *Health and well-being* concerns (health and well-being concerns regarding beneficence [ben HEA], ie, not helping with stress management: mean 3.63, SD 1.51; health and well-being concerns regarding nonmaleficence [nonmal HEA], ie, not increasing stress levels further: mean 4.19, SD 1.62) regarding a dSMI were also moderate. As for the relationships between value-related concerns, significant associations were all positive and small or small to medium in nature (for zero-order correlations, refer to Table S3 in Multimedia Appendix 2).

**Figure 6:**
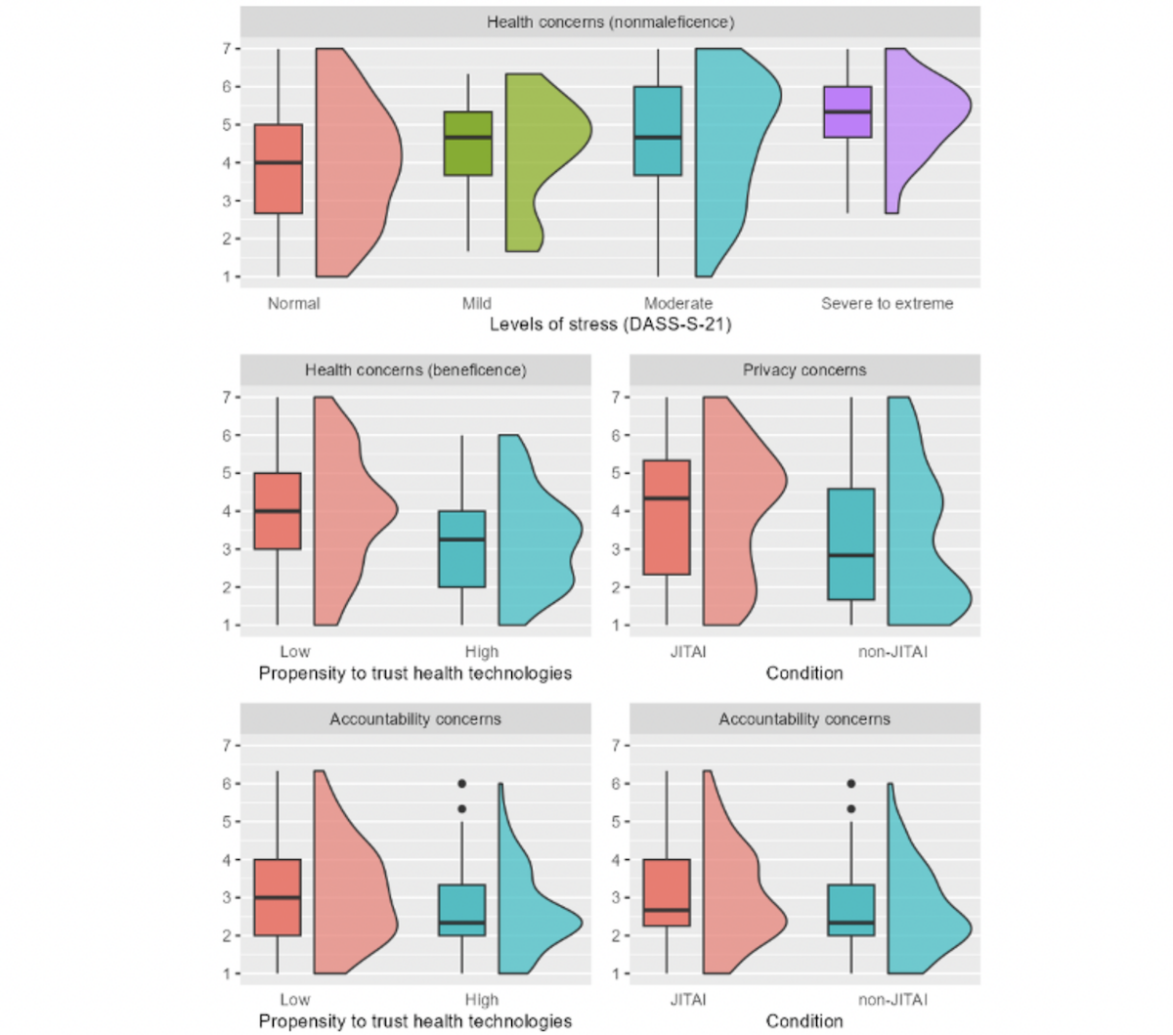
Grouped box plots and distribution plots for value-related concerns that shows significant differences along sample characteristics. DASS-S-21: stress subscale of the Depression Anxiety and Stress Scales; JITAI: just-in-time adaptive intervention.

The median PRI in the participants presented with a JITAI dSMI was 4.33, whereas the median PRI in the participants presented with a non-JITAI dSMI was 2.83. A Mann-Whitney *U* test showed that this difference was statistically significant (JITAI: n=100 and non-JITAI: n=70; *U*=4322.5, *P*=.009, δ=0.24; Figure 6). The median ACC in the participants presented with a JITAI dSMI was 2.67, whereas the median ACC in the participants presented with a non-JITAI dSMI was 2.33. A Mann-Whitney *U* test showed that this difference was statistically significant (JITAI: n=100 and non-JITAI: n=70; *U*=4145, *P*=.04, δ=0.18). The median ACC for the individuals with a low PtT was 3.00, whereas the median for the individuals with a high PtT was 2.33. A Mann-Whitney *U* test showed that this difference was statistically significant (low: n=92 and high: N=78; *U*=4359, *P*=.02, δ*=*0.22). The median ben HEA for the individuals with a low PtT was 4.00, whereas the median ben HEA for the individuals with a high PtT was 3.25. A Mann-Whitney *U* test showed that this difference was statistically significant (low: n=92 and high: n=78; *U*=4576.5, *P*=.002, δ*=*0.28). A Kruskal-Wallis test showed significant differences in the median nonmal HEA for different levels of stress (*H*_3_=10.72, *P*=.01, η^2^=0.05). Post hoc Mann-Whitney *U* tests showed that only the difference between the individuals with normal stress levels (median nonmal HEA 4.00) and the individuals with severe to extremely severe stress levels (median nonmal HEA 5.33) was significant (normal: n=107 and severe to extreme: n=63; *U*=15=434, *P*=.02, δ*=*0.46). No other differences in AUT, PRI, ACC, IDE, ben HEA, or nonmal HEA were found for the remaining sample characteristics (for detailed results, refer to Multimedia Appendix 3).

#### Health Trade-offs

To get a sense of how much the participants were willing to trade off the values *privacy*, *autonomy*, *identity*, and *accountability* for more *health and well-being* benefits, we asked them how much they agreed with the statements presented in Figure 3. The items in Figure 3 are ordered according to the percentage of responses labeled “agree completely” (darkest shade on the right, from highest to lowest percentage). For example, more than 20% of participants were completely willing to let a dSMI track more of their personal data if it increases their health benefits.

#### Value-Related Wishes

This section includes the participants’ ratings of items describing value-related wishes regarding the design, development, and deployment of a dSMI at the workplace. The items in Figure 1 are listed below each value they reflect and are ordered according to the percentage of responses labeled “very important” (darkest shade on the right, from highest to lowest percentage). Regarding the value privacy, for example, around 80% of the participants rated the first item (ie, being able to delete all collected personal data at any time) as “extremely important.”

#### JITAI-Specific Concerns and Wishes

This section includes the participants’ ratings of items describing the concerns and wishes surrounding the use of a JITAI dSMI. The participants were asked how much they agreed with the following statements when imagining the use of a dSMI with a JITAI component at work. The items in Figure 2 are ordered according to the percentage of responses labeled “agree completely” (darkest shade on the right, from highest to lowest percentage). For example, approximately 40% of the participants agreed completely that they want to be able to tell the dSMI with a JITAI component whether they think the feedback is inaccurate (refer to the first item in Figure 2).

#### User Acceptance

Results showed that the mean levels of intention to use (INT; mean 4.60, SD 1.66), perceived usefulness (USE; mean 4.12, SD 1.40), trust (mean 4.25, SD 1.17), and distrust (mean 3.73, SD 1.18) are all situated in the medium range within the possible score range of 1 to 7 (for Cronbach α, see Table S4, and for plots, see Figure S1 in Multimedia Appendix 2). The median INT for the individuals with a low PtT was 4.33, whereas the median INT for the individuals with a high PtT was 5.50. A Mann-Whitney *U* test showed that this difference was statistically significant (low: n=92 and high: n=78; *U*=2099.5, *P*<.001, δ=0.415). The median USE for the individuals with a low PtT was 3.83, whereas the median USE for the individuals with a high PtT was 5.00. A Mann-Whitney *U* test showed that this difference was statistically significant (low: n=92 and high: n=78; *U*=1796.5, *P*<.001, δ*=*0.50). The median USE for the individuals without EXP was 4.00, whereas the median USE for the individuals with EXP was 5.00. A Mann-Whitney *U* test showed that this difference was statistically significant (no EXP: n=129 and EXP: n=41; *U*=1861.5, *P*=.004, δ*=*0.30). The results of the Kruskal-Wallis test revealed that there was a significant difference between the individuals’ stress levels regarding USE (*H*_3_=7.82, *P*=.05, η^2^=0.03). However, post hoc pairwise comparisons did not remain significant after Benjamini-Hochberg correction. The median trust for the individuals with a low PtT was 4.00, whereas the median trust for the individuals with a high PtT was 4.67. A Mann-Whitney *U* test showed that this difference was statistically significant (low: n=92 and high: n=78; *U*=2033, *P*<.001, δ*=*0.43). The median trust for the individuals without EXP was 4.00, whereas the median trust for the individuals with EXP was 4.67. A Mann-Whitney *U* test showed that this difference was statistically significant (no EXP: n=129 and EXP: n=41; *U*=1999.5, *P*=.02, δ*=*0.24). The median distrust for the individuals with a low PtT was 4.00, whereas the median distrust for the individuals with a high PtT was 3.33. A Mann-Whitney *U* test showed that this difference was statistically significant (low: n=92 and high: n=78; *U*=4806, *P*<.001, δ*=*0.34). The median distrust for the individuals without EXP was 4.00, whereas the median distrust for the individuals with EXP was 3.67. A Mann-Whitney *U* test showed that this difference was statistically significant (no EXP: n=129 and EXP: n=41; *U*=3253.5, *P*=.03, δ*=*0.23). No other differences in INT, USE, trust, or distrust were found for the remaining sample characteristics (for detailed results, refer to Multimedia Appendix 3).

#### Willingness to Share Data

To assess how willing the participants were to share different types of data sources to manage their stress and increase their health and well-being with the help of a JITAI, we asked them whether they would share data on subjective stress symptoms, heart activity, keyboard use, computer mouse movements, mental health status, body posture, and eye movements. The items in Figure 4 are ordered according to the percentage of responses labeled “completely willing” (darkest shade on the right, from highest to lowest percentage). For example, >40% of the participants reported that they would not be willing to share data on their eye movements and body posture recorded using a camera on their computer.

### RQ3: FMA of Value-Related Concerns and Wishes

#### Overview

The 2 open questions prompting the participants to share any additional wishes or concerns resulted in a total of 85 text responses from 70 (41.2%) out of 170 participants. We conducted an FMA of these 85 responses to answer RQ3. We found all our identified values (ie, health and well-being, autonomy, privacy, accountability, and identity) as themes in the participants’ statements. In addition to the existing ones*,* new themes emerged from statements of concerns and wishes surrounding a dSMI at the workplace. The new themes comprised the values *integrability*, *digital independence,* and *user-friendliness*. The themes are discussed in the order of frequency of occurrence (exact counts are noted within parentheses) and organized in “key elements.” Inductive thematic saturation [103] was reached, as no new themes emerged after the analysis of 13 of the total 85 responses.

#### Health and Well-being

Wishes and concerns surrounding health and well-being were most often mentioned by the participants (n=37) and often addressed whether a dSMI helped reduce stress or had no impact at all (key element: *effectiveness*). Furthermore, some participants viewed a dSMI as just another gadget that would soon be forgotten and left unused (*just another gadget*). The participants were also concerned that it would exacerbate their stress further owing to increased digital interactions and the potential of being distracted even more (*problem perpetuation*) or that it would not address the sources of stress (*symptom vs cause*):

A dSMI is a useful supplement for relaxation during stress and feeling overwhelmed. But it depends entirely on the willingness of the user. Unfortunately, in this day and age, a dSMI only contributes to the treatment of symptoms and not the causes.

Participant 44

#### Privacy

The participants raised many PRI that covered a range of topics (n=21). Most frequently, the participants were concerned about being surveilled and controlled by their employer if data would be shared with the management (*surveillance*)*.* Similarly, the participants were concerned that others might know that they were using a dSMI if hardware or software wear was visible (*visibility*). The sensitivity of the data itself was also mentioned in relation to a general loss of privacy by having personal and health-related data collected at the workplace (*data sensitivity*). This also included the fear that the data would be shared with health insurers. The participants were also concerned about how and where the data would be stored and whether unauthorized access could be prevented (*data security*):

The data would have to be transferred to the servers of an external and independent company, which has been appropriately certified [in terms of confidentiality and data protection].

Participant 3

#### Autonomy

The participants’ statements about autonomy were multifaceted (n=18). Most often, the concern of becoming dependent on a dSMI was raised (*dependence*). This included feeling a pressure to use it and not being able to manage stress without it, as it might replace an individual’s self-awareness and perception of their symptoms and situation. Moreover, the participants would value that the manner and frequency of interaction with a dSMI are adjustable and personalizable (*control over*). The participants also noted the importance of voluntarily using a dSMI at the workplace rather than being forced by the management (*voluntary use*):

It must not be pushed/advertised/‘rewarded’ too much by the company so that there is no pressure or expectation to use it.

Participant 58

#### Integrability

A new value that emerged from the FMA was that some participants wished for seamless integration of the dSMI into the existing hardware, including desktop computers, laptops, smartphones, smartwatches, and fitness wristbands, rather than having to acquire and use additional equipment (n=13). Moreover, some participants wished to be able to connect the dSMI data with other health-related data from other applications:

I would like it if it were possible to compare biological and psychological mechanisms in a simple way. For example, performance in different cycle phases, sleep quality and stress level, etc.

Participant 98

#### Accountability

With regard to accountability (n=12), the participants often mentioned that data should be transferred to and stored at a known and certified company (*credibility)*. As for testing the effectiveness of a dSMI, the participants had differing views. One of the participants expressed the wish that a dSMI should be broadly tested in all divisions of the company, whereas other participants were concerned that it could be dangerous if vulnerable employees specifically (eg, stressed and at risk for burnout) would be used as guinea pigs to test such a system (*testing scheme*). The participants also wished for the oversight of a dSMI by a medical professional and the incorporation of physician-patient privilege into a dSMI in a credible and trustworthy way (*confidentiality*). Another important aspect raised was that the responsibility for an employee’s well-being should lie with the employer rather than with the employee (*responsibility*). The participants feared that a digital solution such as a dSMI could easily shift the responsibility on the individual:

However, [a dSMI] must not be the only measure [to manage stress]. Otherwise, the responsibility is implicitly placed on the employee, which would be wrong. The employer should bear the main responsibility.

Participant 136

#### Digital Independence

The participants raising concerns surrounding the emerging value we named digital independence mainly expressed that they would not want even more technology and digital interactions dominating their lives (12 counts). Moreover, they thought that a “digital detox” would be the smarter choice to reduce stress rather than an interaction with another digital solution:

In my opinion, digital detox would be a better way of stress relief.

Participant 61

A handful of employees (7/70, 10%) showed resistance or reactance toward using a dSMI at the workplace, reflected in more emotional and extreme statements. For example, one of the participants wrote that they would refuse using a dSMI, even if it meant quitting their job.

#### User-friendliness

In terms of user-friendliness, the participants’ wishes seemed to align (n=9). Namely, they valued a simple and logical design that was not too “overloaded” and used appealing colors (*straightforward design)*. As for functionality, the settings should be designed intuitively and be easily understandable (*easy use*). Moreover, the participants wished for a system that could be deactivated and put on snooze, reminding them to come back for exercises (*adaptability*):

If you click exercises too often, this has to be signaled in some way, otherwise the benefit is lost.

Participant 127

#### Identity

Identity-themed statements were made least often (n=4). The main aspect mentioned by the participants was that a dSMI should be recognized by the employer and that using a dSMI should not lead to any professional or personal disadvantages (*recognition*).

## Discussion

### Overview

In this study, we distilled a set of values implicated in the design, development, and deployment of a dSMI at the workplace from the literature as part of the conceptual investigation of the VSD framework. This was followed by VSD’s empirical investigation of the identified values in the form of a web-based experiment. Here, we used a mixed methods approach to investigate how potential users comprehend the identified values, how a JITAI component affects the importance of these values, and whether novel values emerge from the participants’ responses.

### Sample Characteristics

Among the participants in our study, there were slightly more men (99/170, 58.2%) than women, a ratio that is reflective of company statistics (ie, 59% men), whereas fewer managers (78/170, 45.9%) than regular employees participated, also reflective of the company’s statistics (ie, 42% managers). Regarding age, most employees (89/170, 52.4%) were aged between 30 and 50 years in the sample as well as in the company overall (55%), whereas the employees aged <30 years (study: 27/170, 15.8%; company: 13%) and those aged >50 years (study: 53/170, 31.1%; company: 32%) were similarly represented in numbers. Most participants (144/170, 84.7%) have had experiences with health technologies, especially for fitness and physical activity purposes (133/170, 78.2%). This number is slightly higher than that in other studies exploring attitudes toward mobile health and eHealth; for example, 48.9% of the participants reported having used mobile health in the study by Zia et al [65], whereas 65% of the participants indicated having used health-related apps in the past in [99]. Arguably, employees already experienced with such technologies might have felt more inclined to participate in the study, reflecting a self-selection bias. As for levels of stress (DASS), one-third (48/170, 28.2%) of our participants experienced mild to moderate stress levels, whereas one-tenth (15/170, 8.8%) of our participants experienced severe to extremely severe stress levels in the past week [79,104]. These findings are in line with the results of the 2022 Swiss Job Stress Index, in which 28.2% of Swiss employees were classified as critically stressed [59].

For the FMA, we relied on a subsample of 70 (41.2%) out of 170 participants who provided answers to the open questions regarding additional concerns or wishes. Here, explorative *χ*^2^ tests revealed no significant differences in characteristics (ie, age, gender, position, PtT, DASS, and condition) between the participants who provided free-text responses and those who did not. The relatively low response rate might indicate that, for most participants, the closed items of the survey succeeded in addressing all their major value-related concerns surrounding dSMI at the workplace.

### Employees’ Value-Related Concerns and Wishes

#### Health and Well-being

The participants of our study were highly concerned about the effectiveness of a dSMI and worried that it might even amplify their stress levels, especially the employees who were already with severe to extremely severe stressed (compared with employees with normal levels of stress). Indeed, information, communication, and computer technology as well as digitalization in general can act as sources of stress, also termed “technostress” [105]. Health and well-being are undoubtedly a central value of potential users. Nonetheless, the centrality of health and well-being as a value does not exclude other relevant values in this context that might also conflict with it [54].

#### Privacy

The participants were also highly concerned about privacy, even more so when introduced to a JITAI dSMI. This underlines the importance of privacy as a value, especially when big data are collected and an ML decision system is used [63,106]. Employees might fear discrimination and profiling (eg, helping to decide on who gets promoted and who is let go) if such personal data become identifiable and accessible by the company’s management. The qualitative analysis further revealed that the employees were also concerned about the visibility of the components of a dSMI, which may relate to a phenomenon known as obtrusiveness, a feeling of being watched or surveilled by either colleagues or the management [44,107]. As for data sources, the participants were the least willing to share data on body posture and eye movements to help manage their stress levels and mental health. These findings are in line with the results of another study [98] that found that knowledge workers were more interested in using a wearable device, closely followed by sharing mouse and keyboard use data, rather than being videotaped to track stress levels. Overall, although certain aspects of privacy seem to be nonnegotiable, there is little leeway in designing a dSMI if sharing more data comes with increased health benefits.

#### Autonomy

Western medical ethics views autonomy as the most important ethical principle [108], which was reflected in the number of autonomy-related statements in response to the open questions. By contrast, AUT were rated rather low on the scale of closed questions, although some of the key elements revealed by the FMA were reflected by the closed items of the scale (ie, becoming dependent and feeling pressured). In terms of trading autonomy in exchange for more health benefits, there was a tendency to give up some control over interactions with a dSMI. Professional points of view emphasize that the health care professional is responsible for always balancing out a patient’s autonomy and therapeutic care in the setting of internet- or mobile-supported interventions, although this is challenging [64,109].

#### Identity

IDE were rated rather low and barely mentioned, which seems to contradict the prevailing stigma surrounding mental health issues at the workplace [110-112]. Nevertheless, a handful of employees voiced that their employer should recognize and support the use of a dSMI, which stands somewhat in conflict with the AUT raised by others that a dSMI should not be “pushed” or “advertised too much” by the employer. In terms of trading identity for more health benefits, not all participants were willing to wear more physically visible parts. The visibility of a device or system is related to the values privacy and identity [95]. For example, an employee might be afraid of being treated or seen differently while wearing a visible part of a dSMI. Zia et al [65] also found that the participants were less willing to use a health app that required a visible accessory to manage gastrointestinal symptoms. In conclusion, employees are mainly concerned about their identity in terms of reputation and in relation to others at work rather than in terms of threatening their self-image.

#### Accountability

The individuals who were presented with a JITAI dSMI had significantly higher ACC than those introduced to a non-JITAI dSMI. However, the effect size of the difference was small. Furthermore, the individuals with a low PtT had greater ACC than those with a high PtT. This finding relates to prior research on trust in automation, which found that different levels of PtT affect individual responses to risks and adverse consequences associated with the use of automated systems, such as JITAIs [113]. The qualitative analysis revealed that the responsibility regarding employees’ well-being should not be shifted solely onto the employees and that health professionals should accompany the deployment. Indeed, using the guise of “empowerment of the patient” to shift responsibility from specialists to individuals is a known ethical risk and can increase individuals’ stress levels and mental health issues [114]. When it comes to accountability directly linked to the use of ML models for stress detection, it seems that users just want to know that they received a prompt because of a specific stress level derived from certain data sources but not so much about how the ML model exactly arrived at this output. These results might also suggest that health and well-being, that is, the effectiveness and outcomes of a dSMI, rather than the inner workings of a JITAI, is a priority. This is rather surprising because ML models are known for their opacity and the difficulties in understanding [115] and risk of misunderstanding the information they provide [116].

#### Digital Independence, Integrability, and User-friendliness

Digital independence emerged as a new value from the FMA and is closely related to autonomy and health and well-being. Autonomy-related aspects of digital independence included not wanting to be controlled or dominated by the “digital world” and believing that too much time is spent on the web or spent engaging with a device, the so-called digital overuse [117]. Overusing digital devices leaves users feeling dissatisfied and makes them resort to detox strategies that aim to reduce interaction time and help gain back autonomy and well-being [117,118]. Our study suggests that there is a subgroup of people who are principally not interested in any digital solutions for the management of stress at the workplace, including a small minority that showed some form of resistance or reactance toward using a dSMI at the workplace, in line with the results from other studies regarding feeling tracked at the workplace (eg, Strömberg and Karlsson [119]). Nevertheless, VSD can help create digital well-being technologies that do not promote overuse by design [117].

Finally, integrability and user-friendliness also emerged as new values from the FMA. In particular, limiting the number of different devices required to interact with the dSMI, which was requested by some users, is a way to reduce digital dominance.

### User Acceptance

The perceived intention to use a dSMI and perceived usefulness of a dSMI were moderate to high in the sample at hand, whereas variability was also high. Some of the variability may be explained by the degree of a person’s general PtT and their prior EXP. In a 2021 study, Kallio et al [98] found that 75.5% of the respondents were generally interested in stress level monitoring at work. The qualitative analysis by Lentferink et al [120] also found support for the use of dSMIs at the workplace. In terms of what traits might explain user acceptance, we found that the people with a high PtT had a significantly greater intention to use and higher ratings of usefulness than the people with a low PtT. Our results also suggest that prior EXP increases levels of trust and lowers levels of distrust in a dSMI. This is in line with previous research, which shows that prior experience directly impacts the development of trust [121].

### Deriving Specific Design Requirements

As part of the technical investigation of VSD, we now propose a set of specific requirements on different levels of design, recruitment, and deployment and the everyday use of a dSMI at the workplace based on the results of the conceptual and empirical investigation. Specifically, these design requirements apply to a dSMI meant to be used during working hours, either at the office or at the home office, and not during the employees’ leisure time.

#### Design

In terms of data, the participants’ responses suggest that as little data as possible should be collected (“data minimisation” [120,122]). If possible, a VSD-informed dSMI should compute employees’ stress levels using more unobtrusive data sources. Willingness to share health-related data is also greatly dependent on whom the data are shared with and for what purpose the shared data are used [123]. Here, our results indicate that data should not be shared with third parties, especially with the management, in an identifiable or a nonaggregated manner. Users should always be granted access to their raw data and the option of irreversibly and completely deleting their data. The collected data should be stored and processed locally on their devices as much as possible (ie, client-side processing). For data that must be processed and stored externally, data management should be overseen and executed by a certified third party rather than in company servers. With regard to the system settings of a VSD-informed dSMI, a user should always be able to adjust the degree of privacy, which can include what kinds of, how much, and with whom data are shared. Moreover, the settings regarding stress level feedback and exercises should be personalized and adjustable as much as possible. Although a JITAI may infer optimal opportune times for intervention delivery [124], the amount and time windows of interaction with the intervention should still be controllable. Both prompts and feedback should come with transparent yet easy-to-understand explanations of the predictions and with the option to review the stress level feedback together with an expert if users believe that the stress level predictions are not reflective of their own assessment. They should also adapt to the increasing competence and progress of users to foster their autonomy. Finally, a dSMI should be integrable into existing and commonly used devices as much as possible and run on the available operating systems. The design of the interface should be simple and understandable as well as easy and intuitive to use.

#### Recruitment and Deployment

Recruiting employees at the workplace to use a dSMI should be handled with care. Marketing dSMIs as a well-being technology rather than as a clinical product might carry less stigma because they are not directly associated with a disease or an illness [44]. The company should find the right balance between recognizing and advertising dSMIs and supporting employees without suggesting expectations or coercion to participate. This is important for ensuring employees’ privacy, autonomy, and professional and personal identities. As at least 1 department must be involved in offering a dSMI at the workplace, a VSD-informed dSMI could be provided by an external company with the support of health professionals yet advertised and supported by the human resource department, whose responsibility it is to foster work safety, rather than by the management. This might help mitigate the concern of being surveilled by the management or feeling pressured by the management to use a dSMI. Furthermore, implementing a dSMI companywide can help counter stigmatization [95]. Transparent and complete information that addresses aspects of privacy and accountability (ie, terms of condition or informed consent) should be made available during the recruitment and on-boarding processes and throughout the period of use. This should enable autonomous and independent decision-making and foster the trustworthiness of a dSMI.

#### Use

Employees wished for an intervention that fosters independence, autonomy, and competence. At a higher level, participants wished for an intervention that ensures voluntary use and participation. At a lower level, participants wished for an intervention that ensures control over settings and the system itself by providing a selection of stress management methods to choose from (“one solution does not fit all”) accompanied by clear explanations. Generally, users would want to limit the use of a dSMI as well as interruptions to their daily routine. The system should also provide progress updates and adjust user feedback. Contact with a health professional or technician should be provided in cases of problems, questions, or emergencies. In terms of monitoring and JITAI-specific properties, users want to be able to inform the dSMI that they believe the stress level feedback is wrong and to receive explanations for why an intervention prompt was triggered. Our study also shows that employees are more worried about being labeled as stressed even if they are not (false positives) compared with not being labeled as stressed even if they are (false negatives). Therefore, the ML model predicting stress levels should be designed accordingly to account for this tendency. Finally, concerns regarding privacy and accountability suggest that a JITAI and monitoring component might not be a solution for everyone interested in a dSMI. Therefore, a VSD-informed dSMI should offer the option to deactivate and activate the monitoring and JITAI component and the associated data collection at any time. Furthermore, companies should offer nondigital alternatives to dSMIs for helping their employees manage stress levels to satisfy the value digital independence.

### Limitations

This study comes with a series of limitations. First, we cannot rule out that the participants had differing preconceived ideas and notions of a dSMI, what it does, and what it is capable of (eg, with whom the data are shared, what data are collected, and where they can and should use a dSMI). Consequently, the participants might have drawn different conclusions from the videos and had different understandings of a dSMI. These differences might have affected their user acceptance ratings and the type and extent of concerns they reported. Second, to avoid subconsciously biasing the participants in the non-JITAI dSMI condition, their video did not explicitly declare the absence of any JITAI component in the presented dSMI. Thus, some participants might have imagined a JITAI despite being assigned to the non-JITAI dSMI condition. Although we hopefully identified all these participants through the manipulation check, this design inherently led to a slight imbalance between the 2 experimental conditions. Third, although we believe that we covered the most fundamental ethical issues and concerns applicable to the dSMI context, the list of proposed values that emerged from the literature search and the FMA is a starting point and is neither exhaustive nor complete. Fourth, although we drew from the existing literature and checked the validity and reliability of our items and scales, the survey was self-developed; therefore, more thorough testing is needed to validate these scales. Fifth, we cannot rule out a self-selection bias in our sample. Perhaps we recruited already technology-affine individuals and more suspicious and critical employees rather than the moderate center. The recruitment pool was also limited to a single Swiss insurance company, which might further limit the generalizability of our results. Finally, although the post hoc power of medium effect sizes lies in the acceptable to desired range of 0.75 and 0.80, the post hoc power of small effect sizes was around 0.30. These estimates warrant further research with larger samples to replicate and validate our findings, especially because this is the first study to apply VSD in the context of dSMIs at the workplace.

### Conclusions

VSD integrates and respects the social aspect of a sociotechnical system, such as a dSMI, by providing a framework that guides the translation of values into specific design requirements. To the best of our knowledge, this is the first study to apply VSD to the context of a dSMI at the workplace. Through VSD, we identified the main values of a dSMI at the workplace (ie, health and well-being, privacy, autonomy, accountability, and identity). Overall, there is a general interest and willingness to try out and use dSMIs among employees. In terms of value-related wishes and concerns, employees’ responses emphasized the importance of deploying a voluntary, effective, and easy-to-use dSMI that does not increase “technostress,” especially in at-risk employees; can be held accountable; and protects users’ privacy, even more so when a dSMI includes the collection and processing of biomedical big data. As there is also a minority of employees who are wary of being dominated by technologies in general, we recommend providing employees with a nondigital offer for managing stress. Taken together, the results of this study and our research protocol promote the VSD of digital health interventions aimed at managing work-related stress in an actionable way.

## Appendix

Multimedia Appendix 1 Detailed information on the literature search.

Multimedia Appendix 2 Value-related and user acceptance scales.

Multimedia Appendix 3 Comprehensive overview of the quantitative results.

## Abbreviations

ACC: accountability concerns
AI: artificial intelligence
AUT: autonomy concerns
ben HEA: health and well-being concerns regarding beneficence
DASS: Depression, Anxiety, and Stress Scales
dSMI: digital stress management intervention
EXP: experience with health technologies
FMA: framework method analysis
IDE: identity concerns
INT: levels of intention to use
JITAI: just-in-time adaptive intervention
ML: machine learning
nonmal HEA: health and well-being concerns regarding nonmaleficence
PRI: privacy concerns
PtT: propensity to trust health technologies
RQ: research question
SMI: stress management intervention
VSD: value sensitive design
