# Supplementary material for: Investigating Employees’ Concerns and Wishes Regarding Digital Stress Management Interventions With Value Sensitive Design: Mixed Methods Study
Source: J Med Internet Res. 2023 Apr 13;25:e44131. doi: 10.2196/44131 (PMC10141316; doi:10.2196/44131)
Supplement: Multimedia Appendix 1 [file jmir_v25i1e44131_app1.docx]

This is a Multimedia Appendix to a full manuscript published in the J Med Internet Res. For full copyright and citation information see [http://dx.doi.org/10.2196/jmir.](http://dx.doi.org/10.2196/jmir.xxxx)44131

## Multimedia Appendix: Literature Search

### Detailed Information on Literature Search

We ran a search of publications on the databases Web of Science and SCOPUS using a combination of terms related to *ethics* (ethic* OR moral* OR value), *technology* (“internet of things” OR IoT OR “ubiquitous computing” OR wearable* OR “big data” OR technology OR comput* OR “artificial intelligence” OR “machine learning” OR mobile OR digital OR “recommend* system*”), *health* (health* OR medic* OR bio* OR wellbeing OR well-being OR stress), the relevant *domain* (work OR workplace* OR employ* OR occupation*), *monitoring* (“health monitoring” OR “personal health monitoring” OR “somatic surveillance” OR “health surveillance” OR “detection” OR “ecological momentary assessment*” OR EMA*) and *interventions* (intervention* OR “just-in-time intervention*” OR “just-in-time adaptive intervention*” OR JITAI* OR “intervention prompt*”). The search was carried out from February to April 2021 and included English and German publications from the year 2012 until 2021.

Table S1. The twenty-three selected articles from the literature search in alphabetical order of authors.

| Reference | Extracted values, principles, and ethical criteria |
| --- | --- |
|  |  |
| *Principles of biomedical ethics* [60] | Respect for autonomy, non-maleficence, beneficence, justice |
| *Ethical aspects of digital health from a justice point of view* [58] | Justice, autonomy, privacy, security, trust, transparency, accountability, inclusiveness |
| *The ethics of digital well-being: a thematic review* [49] | autonomy and self-determination, privacy |
| *Dataveillance and information privacy concerns: ethical and organisational considerations* [66] | Privacy, justice, fairness |
| *Ethical and legal challenges of artificial intelligence in nuclear medicine* [67] | Beneficence, non-maleficence, fairness and justice, safety, reliability, security, privacy and confidentiality, mitigation of bias, transparency and visibility, explainability and comprehensibility, autonomy, judgement, and decision-making, collegiality, accountability, governance, inclusiveness |
| *Ethical considerations of using machine learning for decision support in occupational health: an example involving periodic workers' health assessments* [63] | *Beauchamp & Childress’ principles* |
| *Value sensitive design and information systems [91]* | *Human welfare, ownership and property, privacy, freedom from bias, universal usability, trust, autonomy, informed consent, accountability, courtesy, identity, calmness and environmental sustainability.* |
| *Participatory disease surveillance systems: ethical framework* [[68]](https://paperpile.com/c/f0gPo9/FcRA) | *Beauchamp & Childress’ principles* |
| *Real-world data to generate evidence about healthcare interventions: the application of an ethics framework for big data in health and research* [62] | Integrity, public benefit, reflexivity, transparency, accountability |
| *Advancing public health in the age of big data: methods, ethics, and recommendations* [69] | Privacy and misuse of data, informed consent and transparency, risk associated with discrimination and inaccurate prediction |
| *Recommender systems and their ethical challenges* [70] | Privacy, autonomy, identity |
| *The ethical implications of personal health monitoring* [34] | Privacy, autonomy, obtrusiveness and visibility, stigma and identity, medicalisation, social isolation, delivery of care, and safety and technological need |
| *Designing the health-related internet of things: ethical principles and guidelines* [71] | User engagement, non-maleficence and beneficence, respect autonomy, respect individual privacy, respect group privacy, inclusiveness and diversity, data minimisation, trust and confidentiality, transparency and accountability |
| *Ethics of the health-related internet of things: narrative review* [44] | Privacy, trust, autonomy, ownership, data access |
| *Psychiatric advance directives and artificial intelligence: a conceptual framework for theoretical and ethical principles* [72] | Loyalty, vigilance, *Beauchamp & Childress’ principles* |
| *Investigating ethical design requirements for digitalized healthcare support: the case of ambulatory physiotherapeutic assistance systems* [54] | Autonomy, competence, privacy and security, design for all |
| *Building the case for actionable ethics in digital health research supported by artificial intelligence* [15] | Respect for persons, beneficence, justice, participant privacy, access and usability |
| *‘My Fitbit thinks I can do better!’ Do health promoting wearable technologies support personal autonomy?* [73] | Autonomy |
| *Designing for motivation, engagement and wellbeing in digital experience* [61] | Autonomy, relatedness, competence |
| *Ethical considerations for digitally targeted public health interventions* [74] | Autonomy, privacy, expectation bias |
| *Machine learning and artificial intelligence research for patient benefit: 20 critical questions on transparency, replicability, ethics, and effectiveness* [75] | Transparency, reproducibility, ethics, and effectiveness |
| *An ethics framework for big data in health and research* [35] | Harm minimisation, integrity, justice, liberty/autonomy, privacy, proportionality, public benefit, solidarity, stewardship, accountability, consistency, engagement, reasonableness, reflexivity, transparency, trustworthiness |
| *Download alert: understanding gastroenterology patients' perspectives on health-related smartphone apps* [65] | Usability, feasibility, credibility, obtrusiveness |

### 
