# Supplementary material for: Investigating Employees’ Concerns and Wishes Regarding Digital Stress Management Interventions With Value Sensitive Design: Mixed Methods Study
Source: J Med Internet Res. 2023 Apr 13;25:e44131. doi: 10.2196/44131 (PMC10141316; doi:10.2196/44131)
Supplement: Multimedia Appendix 2 [file jmir_v25i1e44131_app2.docx]

This is a Multimedia Appendix to a full manuscript published in the J Med Internet Res. For full copyright and citation information see [http://dx.doi.org/10.2196/jmir.](http://dx.doi.org/10.2196/jmir.xxxx)44131

## Multimedia Appendix: Value-related and User Acceptance Scales

Table S1. Individual items of the trust, distrust, intention to use and usefulness scales*.*

|  | Item | Sources |
| --- | --- | --- |
|  |  |  |
| **Intention to Use** | | Technology Acceptance Model [[76, 102]](https://paperpile.com/c/f0gPo9/Bgd9g) |
|  | 1. Given the opportunity, I would like to use a dSMI. |  |
|  | 1. I would use a dSMI regularly. |  |
|  | 1. I would give the use of a dSMI a try. |  |
| **Usefulness** | | Technology Acceptance Model [[76, 102]](https://paperpile.com/c/f0gPo9/Bgd9g) |
|  | 1. Using a dSMI would make me cope with stress. |  |
|  | 1. Using dSMI would give me confidence in my stress management. |  |
|  | 1. Overall, a dSMI is useful. |  |
| **Trust** | | Trust in Automation Scale [77]; German version: [78] |
|  | 1. The digital stress management intervention (dSMI) is dependable. |  |
|  | 1. The dSMI is reliable. |  |
|  | 1. I can trust the dSMI. |  |
| **Distrust** | | Trust in Automation Scale [77]; German version: [78] |
|  | 1. The dSMI behaves in an underhanded manner. |  |
|  | 1. I am suspicious of the dSMI’s intent, action, or outputs. |  |
|  | 1. I am wary of the dSMI. |  |

### Confirmatory Factor Analysis of Value-related Concerns Scales

The confirmatory factor analysis of the six scales assessing value-related concerns (i.e., HEA_ben_, HEA_non-mal_, PRI, AUT, IDE, ACC) based on 170 observations. Since multivariate normal distribution of the scales was not met, we use the robust estimator MLR. MLR was used to estimate model parameters and goodness-of-fit of the CFA model was examined with RMSEA<0.05, SRMR<0.068, CFI>0.95, and TLI>0.95. Additionally, the normed chi-square/df ratio ≤3 rule (normed chi-square) was used. The mode chi-square was significant, χ2(113)=201.15, *P*<.001. Regarding model fit, our robust model showed an acceptable fit, except for the RMSEA, and factor loadings from 0.52 to 0.97. Robust goodness-of-fit indices of the model are as follows: RMSEA=0.07, SRMR=0.08, CFI=0.93, TLI=0.91 and normed chi-square=1.78. In addition, all factor loadings are above 0.30 and significantly load on the respective factor (all z’s<2.92, all *P*’s<.01).

Table S2. Results of confirmatory factor analysis of value-related concerns regarding a digital stress management intervention (dSMI) at the workplace. All items could be answered on a 7-point Likert scale.

|  | Item | Factor loading |
| --- | --- | --- |
|  |  |  |
| **Health and well-being regarding beneficence (HEA_ben_)** | |  |
|  | How true are the following concerns for you when imagining the use of a dSMI at work? |  |
|  | 1. A dSMI would not really help me with my stress. | .97 |
|  | 1. A dSMI would not really help me with my mental health. | .82 |
| **Health and well-being regarding non-maleficence (HEA_non-mal_)** | |  |
|  | I am concerned that a dSMI could increase my stress levels because: |  |
|  | 1. It could send recommendations for exercise sessions at an inopportune moment. | .77 |
|  | 1. It might provide too much information for me to be able to process. | .84 |
|  | 1. It might take too much time to use it. | .78 |
| **Privacy (PRI)** | |  |
|  | 1. I would be afraid that my personal data collected by a dSMI would get into the “wrong hands”. | .77 |
|  | 1. I would worry that my data collected by a dSMI was shared with the management of my company even in an anonymized and aggregated form. | .90 |
|  | 1. I would be concerned that others would ask me about my stress level and intervention progress. | .79 |
| **Autonomy (AUT)** | |  |
|  | How true are the following concerns for you when imagining the use of a dSMI at work? |  |
|  | 1. I worry that by using a dSMI I would become dependent on it to manage my stress. | .93 |
|  | 1. I am concerned that I would feel pressured to use a dSMI more often than I would like. | .69 |
|  | 1. I worry I would have to carry too much responsibility managing my stress only with a dSMI instead of being guided by a health professional. | .73 |
| **Identity (IDE)** | |  |
|  | 1. I worry that my colleagues would view me as weak or ill if they knew I used a dSMI. | .92 |
|  | 1. I worry that my superiors would view me as weak or ill if they knew I used a dSMI. | .88 |
|  | 1. I would view myself as weak or ill if I used a dSMI | .79 |
| **Accountability (ACC)** | |  |
|  | 1. I worry that the dSMI is exclusively digital and does not include personal contact with health professionals. | .52 |
|  | 1. I worry that no one could be held accountable if something with a dSMI went wrong. | .68 |
|  | 1. I worry that a dSMI would overpromise its health benefits | .71 |

Table S3. Means, standard deviations and correlation analysis (Pearson *r* and *P* value) among health concerns (HEA_ben_ and HEA_non-mal_), privacy (PRI), autonomy (AUT), identity (IDE), and accountability (ACC) concerns.

|  | *Mean* | *SD* | HEA_ben_ | HEA_non-mal_ | PRI | AUT | IDE | ACC |
| --- | --- | --- | --- | --- | --- | --- | --- | --- |
|  |  |  |  |  |  |  |  |  |
| **HEA_ben_** | 3.63 | 1.51 |  |  |  |  |  |  |
|  |  |  | (n.a.)^b^ |  |  |  |  |  |
| **HEA_non-mal_** | 4.19 | 1.62 |  |  |  |  |  |  |
| *r* |  |  | 0.47 | (.84)^a^ |  |  |  |  |
| *P* value |  |  | <.001 |  |  |  |  |  |
| **PRI** | 3.69 | 1.81 |  |  |  |  |  |  |
| *r* |  |  | 0.14 | 0.19 | (.85)^a^ |  |  |  |
| *P* value |  |  | 0.07 | 0.01 |  |  |  |  |
| **AUT** | 2.63 | 1.32 |  |  |  |  |  |  |
| *r* |  |  | 0.25 | 0.50 | 0.32 | (.72)^a^ |  |  |
| *P* value |  |  | .001 | <.001 | <.001 |  |  |  |
| **IDE** | 2.14 | 1.37 |  |  |  |  |  |  |
| *r* |  |  | 0.22 | 0.17 | 0.27 | 0.39 | (.85)^a^ |  |
| *P* value |  |  | .004 | .03 | <.001 | <.001 |  |  |
| **ACC** | 2.91 | 1.24 |  |  |  |  |  |  |
| *r* |  |  | 0.30 | 0.45 | 0.52 | 0.45 | 0.37 | (.66)^a^ |
| *P* value |  |  | <.001 | <.001 | <.001 | <.001 | <.001 |  |
| ^a^ Cronbach α coefficients are reported along the diagonal in parentheses where applicable.  ^b^ Not applicable. | | | | | | | | |

Table S4. Means, standard deviations and Cronbach α’s for user acceptance measures.

|  | *Mean* | *SD* | α |
| --- | --- | --- | --- |
|  |  |  |  |
| Intention to use | 4.60 | 1.66 | .94 |
| Usefulness | 4.12 | 1.40 | .93 |
| Trust | 4.25 | 1.17 | .87 |
| Distrust | 3.73 | 1.18 | .67 |


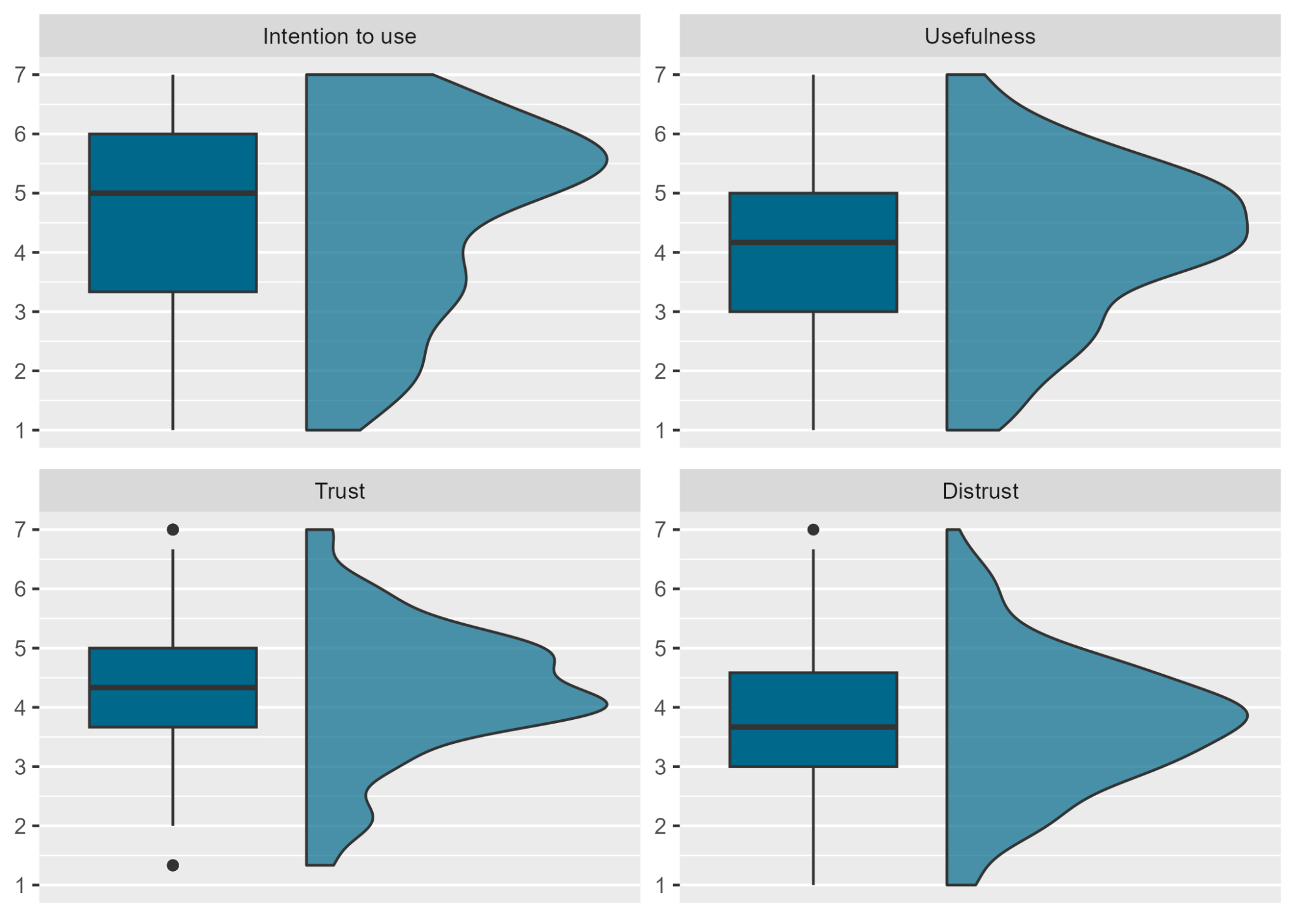


Figure S1. Boxplots and distribution plots of user acceptance measures.
