# Supplementary material for: Investigating Employees’ Concerns and Wishes Regarding Digital Stress Management Interventions With Value Sensitive Design: Mixed Methods Study
Source: J Med Internet Res. 2023 Apr 13;25:e44131. doi: 10.2196/44131 (PMC10141316; doi:10.2196/44131)
Supplement: Multimedia Appendix 3 [file jmir_v25i1e44131_app3.docx]

This is a Multimedia Appendix to a full manuscript published in the J Med Internet Res. For full copyright and citation information see [http://dx.doi.org/10.2196/jmir.](http://dx.doi.org/10.2196/jmir.xxxx)44131

## Multimedia Appendix: Comprehensive Overview of Quantitative Results

Table S1. Results of non-parametric t- and F-tests of value-related concerns across sample characteristics (N = 170).

|  | Variables | Test statistic | *P* value |
| --- | --- | --- | --- |
|  |  |  |  |
| **Health regarding beneficence** | |  |  |
|  | Age | *F*=0.79 | .85 |
|  | Gender | *U*=3857 | .28 |
|  | Position | *U*=4033.5 | .16 |
|  | Propensity to trust health technologies | *U*=4576.5 | .002 |
|  | Experience with health technologies | *U*=2907.5 | .33 |
|  | Levels of stress | *F*=5.11 | .16 |
|  | Condition | *U*=3401.5 | .75 |
| **Health regarding non-maleficence** | |  |  |
|  | Age | *F*=2.442 | .49 |
|  | Gender | *U*=3514.5 | 1.000 |
|  | Position | *U*=3510 | .81 |
|  | Propensity to trust | *U*=3937 | .27 |
|  | Experience with health technologies | *U*=2681.5 | .89 |
|  | Levels of stress | *F*=10.73 | .01 |
|  | Condition | *U*=3495.5 | .99 |
| **Privacy** | |  |  |
|  | Age | *F*=0.476 | .92 |
|  | Gender | *U*=3381.5 | .67 |
|  | Position | *U*=3831 | .45 |
|  | Propensity to trust | *U*=4119 | .10 |
|  | Experience with health technologies | *U*=2595 | .86 |
|  | Levels of stress | *F*=1.83 | .61 |
|  | Condition | *U*=4322.5 | .01 |
| **Autonomy** | |  |  |
|  | Age | *F*=6.392 | .09 |
|  | Gender | *U*=3420.5 | .77 |
|  | Position | *U*=4186.5 | .06 |
|  | Propensity to trust | *U*=3722.5 | .67 |
|  | Experience with health technologies | *U*=2867.5 | .41 |
|  | Levels of stress | *F*=1.75 | .63 |
|  | Condition | *U*=4028.5 | .09 |
| **Identity** | |  |  |
|  | Age | *F*=0.20 | .98 |
|  | Gender | *U*=3190.5 | .29 |
|  | Position | *U*=3263 | .29 |
|  | Propensity to trust | *U*=3386 | .51 |
|  | Experience with health technologies | *U*=2342.5 | .26 |
|  | Levels of stress | *F*=3.41 | .33 |
|  | Condition | *U*=3495 | .99 |
| **Accountability** | |  |  |
|  | Age | *F*=1.08 | .78 |
|  | Gender | *U*=3376 | .66 |
|  | Position | *U*=3580.5 | .98 |
|  | Propensity to trust | *U*=4359 | .02 |
|  | Experience with health technologies | *U*=2976.5 | .23 |
|  | Levels of stress | *F*=1.79 | .62 |
|  | Condition | *U*=4145 | .04 |

Table 2. Results of non-parametric t- and F-tests of user acceptance across sample characteristics (N = 170).

|  | Variables | Test statistic | *P* value |
| --- | --- | --- | --- |
|  |  |  |  |
| **Intention to use** | |  |  |
|  | Age | *F*=1.58 | .66 |
|  | Gender | *U*=3664.5 | .64 |
|  | Position | *U*=3654 | .84 |
|  | Propensity to trust health technologies | *U*=2099.5 | <.001 |
|  | Experience with health technologies | *U*=2118.5 | .05 |
|  | Levels of stress | *F*=5.49 | .14 |
|  | Condition | *U*=3404 | .76 |
| **Perceived usefulness** | |  |  |
|  | Age | *F*=4.23 | .24 |
|  | Gender | *U*=3752.5 | .45 |
|  | Position | *U*=4162.5 | .07 |
|  | Propensity to trust | *U*=1796.5 | <.001 |
|  | Experience with health technologies | *U*=1861.5 | .004 |
|  | Levels of stress | *F*=7.82 | .05 |
|  | Condition | *U*=3417.5 | .79 |
| **Trust** | |  |  |
|  | Age | *F*=4.75 | .19 |
|  | Gender | *U*=3906.5 | .21 |
|  | Position | *U*=3937.5 | .27 |
|  | Propensity to trust | *U*=2033 | <.001 |
|  | Experience with health technologies | *U*=1999.5 | .02 |
|  | Levels of stress | *F*=1.56 | .67 |
|  | Condition | *U*=3301.5 | .53 |
| **Distrust** | |  |  |
|  | Age | *F*=3.100 | .38 |
|  | Gender | *U*=3025.5 | .12 |
|  | Position | *U*=3242 | .28 |
|  | Propensity to trust | *U*=4806 | <.001 |
|  | Experience with health technologies | *U*=3253.5 | .03 |
|  | Levels of stress | *F*=0.68 | .88 |
|  | Condition | *U*=3889 | .22 |

## 
